# Efficient production of cynomolgus monkeys with a toolbox of enhanced assisted reproductive technologies

**DOI:** 10.1038/srep25888

**Published:** 2016-05-13

**Authors:** Yunhan Ma, Jiayu Li, Ge Wang, Qiong Ke, Sien Qiu, Liang Gao, Haifeng Wan, Yang Zhou, Andy Peng Xiang, Qunshan Huang, Guoping Feng, Qi Zhou, Shihua Yang

**Affiliations:** 1 College of Veterinary Medicine, Guangdong Provincial Key Laboratory of Prevention and Control for Severe Clinical Animal Diseases, South China Agricultural University, Guangzhou 510642, P. R. China; 2 Shenzhen Key Lab of Neuropsychiatric Modulation and Collaborative Innovation Center for Brain Science, CAS Center for Excellence in Brain Science, Shenzhen Institutes of Advanced Technology, Chinese Academy of Sciences, Shenzhen 518055, P. R. China; 3 Center for Stem Cell Biology and Tissue Engineering, Key Laboratory for Stem Cells and Tissue Engineering of Ministry of Education, Sun Yat-sen University, Guangzhou 510080, P. R. China; 4 Blooming-spring biotechnology development Co., Ltd., of Guangdong, Guangzhou 510940, P. R. China; 5 State Key Laboratory of Reproductive Biology, Institute of Zoology, Chinese Academy of Sciences, Beijing 100101, P. R. China; 6 McGovern Institute for Brain Research, Department of Brain and Cognitive Sciences, Massachusetts Institute of Technology, Cambridge, MA 02139, USA

## Abstract

The efficiency of assisted reproductive technologies (ARTs) in nonhuman primates is low due to no screening criterions for selecting sperm, oocyte, and embryo as well as its surrogate mothers. Here we analyzed 15 pairs of pregnant and non-pregnant cynomolgus monkeys, each pair of which received embryos from one batch of fertilized oocytes, and found ratio of endometrial to myometrial thicknesses in abdominal ultrasonic transverse section of uterus is a reliable indicator for selection of recipients for embryo transfer. We performed 305 ovarian stimulations in 128 female cynomolgus monkeys and found that ovarian stimulation can be performed in a whole year and repeated up to six times in the same monkey without deteriorating fertilization potential of eggs until a poor response to stimulation happened. Fertilization can be efficiently achieved with both conventional and piezo-driven intracytoplasmic sperm injection procedures. In semen collection, semen quality is higher with the penile robe electrical stimulus method compared with the rectal probe method. Moreover, caesarean section is an effective strategy for increasing baby survival rates of multiple pregnancies. These findings provide a practical guidance for the efficient use of ARTs, facilitating their use in genetic engineering of macaque monkeys for basic and translational neuroscience research.

A major milestone in biomedical research was the generation of the first gene knockout mouse through embryonic stem cell based gene targeting^1^, which opened the door for the creation and application of genetically modified animals for breakthrough researches into the basic medical science. In recent years, the emergence of new gene editing technologies, such as zinc-finger nucleases^2^,^3^, transcription activator-like effector nucleases^4^ and CRISPR/Cas9^5^,^6^, makes it possible to achieve gene modification in animal embryos instead of using totipotent embryonic stem cells. It is now possible and attractive to produce gene-modified non-human primate animals for human disease research^7^,^8^,^9^,^10^, especially for diseases with strong genetic contributions, such as autism spectrum disorder, schizophrenia and metabolic diseases.

Assisted reproductive technologies (ARTs) are essential for the production of genetically modified monkeys. Since the birth of the first *in vitro* fertilized rhesus monkey baby^11^, ARTs in rhesus monkeys^12^, cynomolgus monkeys^13^, marmosets^14^ and other primate species^15^ have been established. However, the multi-step, complex procedures and high cost involved in ARTs often result in low efficiency of reproduction in monkeys, which implies insufficient research of reproductive biology and impedes the creation of genetically modified monkeys.

Cynomolgus monkey is an attractive primate animal for genetic engineering of disease models due to its year-round reproductive capability, moderate size, complex behaviors and similarity to humans in physiology and pathology. Currently, there are few reproductive researches in cynomolgus monkeys, which result in a slow development of its ARTs. In this study, we systematically compared several key procedures in ARTs including stimulated ejaculation, seasonal variation of reproduction, superovulation, intracytoplasmic sperm injection (ICSI), embryo transfer and cesarean section. These results enhance the efficiency of ARTs and should increase the success of generating genetically modified monkeys, which helps for the creation of advanced animal models to make key breakthroughs on primate neuropathic diseases research.

## Results

### Comparison of electro-stimulation ejaculation methods

**Figure 1:**
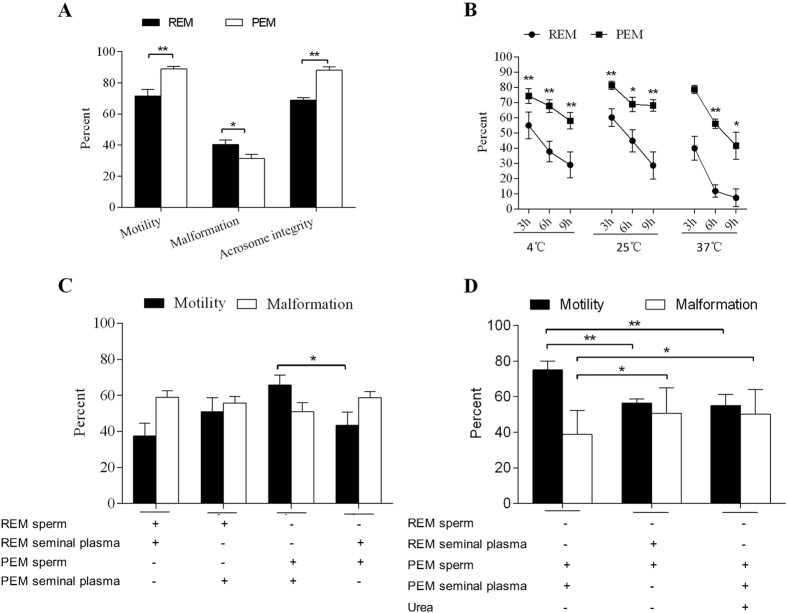
Comparison of Features of Semen Collected by Rectal Probe Electrical Stimulus Method (REM) and Penile Probe Electrical Stimulation Method (PEM). (**A**) Comparison of motility, malformation and acrosome integrity of sperm in semen from REM and PEM, held at 37 °C for 30 min. (**B**) Comparison of motility of sperm in semen between REM and PEM, held at different temperatures for different durations. (**C**) Comparison of motility and malformation after exchanging sperm and seminal plasma between REM and PEM. (**D**) Comparison of motility and malformation after adding urea to seminal plasma. In (**C**,**D**), symbols “+” means existence and “−” is non-existence of semen components in each row. In addition, “+” for urea in (**D**) means urea added to a concentration similar to that in REM seminal plasma. Results are means ± s.e.m. Data are from four independent experiments. Statistical analysis was performed by one-way analysis of variance. *P < 0.05, **P < 0.01.

Eight adult male monkeys were used in this study. Four males were randomly selected to treat with REM and the rest four males were treated with PEM. Each animal received four stimulations at four day intervals. Then, the stimulus methods were exchanged between the two groups and each animal received another four stimulations with an interval of four days. Although REM took less time to achieve ejaculation, PEM was better in semen characteristics including seminal weight, sperm concentration (Table 1), motility (Fig. 1A), rate of intact acrosomes (Fig. 1A and Supplementary Fig. S1A) and malformation rate (Fig. 1A and Supplementary Fig. S1B). Furthermore, sperm motility in PEM semen was higher than that in REM semen when balanced for 3 h, 6 h, and 9 h, at 4 °C, 25 °C and 37 °C, respectively (Fig. 1B). To evaluate the influence of both ejaculation methods on sperm motility, sperm cells and seminal plasma were exchanged between REM and PEM semen, and our results showed that REM sperm became more vital after being mixed with PEM seminal plasma while PEM sperm were less vital when suspended in REM seminal plasma (Fig. 1C). It has been reported that urea in semen critically affects sperm motility^16^. To test if semen was contaminated with urine during collection, measurement of urea concentration in seminal plasma was established (Supplementary Fig. S2A). Then we found that the concentration of urea in seminal plasma from REM was higher than that from PEM (Supplementary Fig. S2B). To further confirm that urea caused low sperm vitality in REM semen, we reconstituted semen in three groups, group 1: PEM sperm + PEM seminal plasma; group 2: PEM sperm + REM seminal plasma; and group 3: PEM sperm + PEM seminal plasma with urea added to the concentration similar to that in REM seminal plasma. Reconstituted semen was held for 30 min at 37 °C, then sperm motility was measured. We found that sperm motility in group 2 was similar to that in group 3 and lower than that in group 1 (Fig. 1D). Taken together, these data suggest that high sperm motility from PEM was probably due to less urea contamination.

### Efficiency of obtaining oocytes by repeated ovarian stimulation

**Figure 2:**
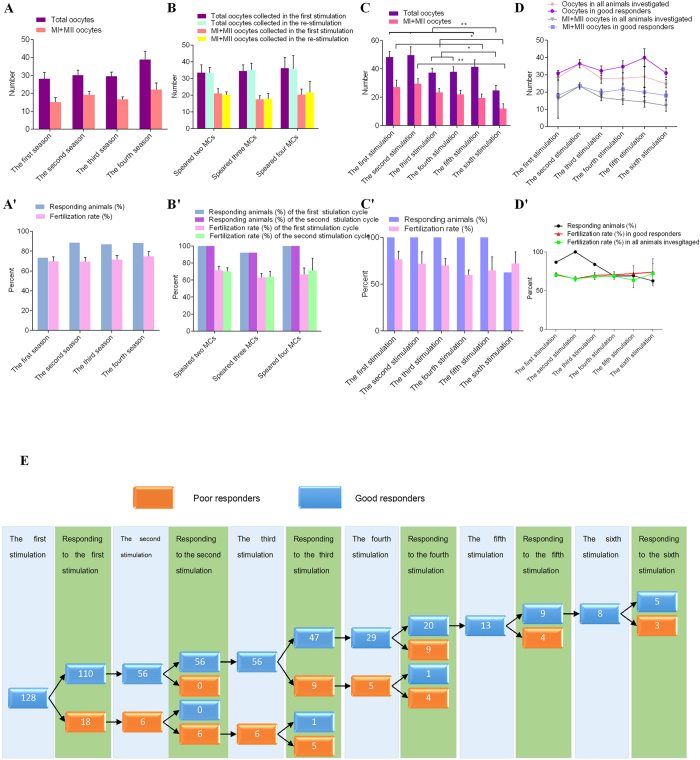
Repeated Ovarian Stimulation in Cynomolgus Monkeys. (**A**,**A’**) The number of oocytes recovered and the proportion of good responders and fertilization rate at the first ovarian stimulation cycle in one whole year. Fertilization was achieved with conventional ICSI. All data are means ± s.e.m. with n = 128 for four quarters. Statistical comparisons were made among the four quarters with one-way analysis of variance with least-significant difference. (**B**,**B’**) The number of oocytes recovered, the proportion of responding animals and the fertilization rate at the first stimulation and the second (restimulation) separated at intervals of two, three and four MCs, respectively. Data are means ± s.e.m. with n = 30 for the three groups. Statistical comparisons were made among the three groups with a generalized linear model repeated-measures. (**C**,**C’**) The numbers of oocytes recovered and responding animals, and the fertilization rate, in cynomolgus monkeys at one to six consecutive, repeated ovarian stimulations at intervals of two to four MCs. Data are means ± s.e.m. with n = 8. Statistical comparison was made among different repeated stimulations with generalized linear model repeated-measures. *P < 0.05. (**D**,**D’**) and (**E**) Data are represented in a schematic drawing of good and poor responders randomly selected for repeated ovarian stimulation and their responses. MC, menstrual cycle.

Cynomolgus monkeys can breed throughout the year^17^. To investigate whether there is no seasonal effect on ovarian response to ovarian stimulation, a total of 128 adult cynomolgus monkeys were randomly allocated into four groups (based on the four seasons in a year) and underwent the same ovarian stimulation protocol (Supplementary Fig. S3). The proportion of good responders and the number of oocytes recovered from good responders did not differ among the four groups. We further found that the fertilization rate of retrieved oocytes did not differ among the four groups (Fig. 2A,A’). Therefore, ovarian stimulation can be efficiently performed year-round in cynomolgus monkeys with similar efficiency.

The length of the menstrual cycle during ovarian stimulation became shorter and the first menstrual cycle after ovarian stimulation became longer compared to subsequent menstrual cycles, as well as those menstrual cycles before ovarian stimulation (Supplementary Fig. S4). In our previous study on rhesus monkeys, repeated ovarian stimulations were performed separated with two menstrual cycles^18^. Here we further tested whether interval length between ovarian stimulations affect oocyte quality. Cynomolgus monkeys (*n* = 30), after the first ovarian stimulation, were randomly divided into three groups for the second stimulation with the same regimen separated at intervals of two, three or four menstrual cycles, respectively (Supplementary Fig. S5). We found that the proportion of good responders, number of oocytes retrieved and fertilization rates were similar among the three groups (Fig. 2B,B’).

Another important consideration is how many times ovarian stimulation can be repeated on an adult female monkey before ovarian response decreases significantly. To explore this question, eight monkeys were consecutively stimulated six times with the same regimen separated at intervals of two to four menstrual cycles. The results indicated that after the first two stimulations, there is a decreasing trend in the proportion of good responders and in the number of oocytes retrieved with increasing times of ovarian stimulations. In particular, the number of retrieved oocytes significantly decreased at the sixth ovarian stimulation compared with that at the first stimulation (Fig. 2C). However, the fertilization rate did not differ among the six stimulations (Fig. 2C’).

As we previously reported^19^, poor responders to ovarian stimulation were encountered in approximately 15–20% either to the first or repeated ovarian stimulations. Here we conducted the first ovarian stimulation in 128 cynomolgus monkeys and found that 110 monkeys (85.9%) responded well while the other 18 monkeys (14.1%) responded poorly (Fig. 2E). We then randomly selected 56 monkeys from the 110 good responders and 6 monkeys from the 18 poor responders for the second ovarian stimulation. Interestingly, the good responders responded well to the second stimulation and the poor responders responded poorly. Subsequently, all of these monkeys were given the third stimulation. Among the 56 good responders, 47 remained good responders and 9 had poor responses. Among the six poor responders, only one was a good responder. Finally, for the eight good responders undergoing the sixth stimulation, five showed good responses and three poor responses. Together, our findings demonstrate that in cynomolgus monkeys, ovarian stimulation can be repeated six times if good responses were achieved at previous stimulation. (Fig. 2D,D’) That is to say that ovarian stimulation should not be repeated any more whenever the monkey shows a poor response to stimulation. On this schema, good ovarian response rate was 88% for first three stimulations and 69% for the fourth and fifth stimulations and 63% for the sixth stimulation.

### Efficiency of conventional and piezo-driven ICSI

Both conventional and piezo-driven ICSI were successful for *in vitro* fertilization in monkeys and differences in fertilization rate and subsequent embryo development were reported^20^,^21^. In the present study, a total of 179 mature oocytes from cynomolgus monkeys were pooled and randomly allocated to undergo conventional or piezo-driven ICSI. Our results demonstrate that both ICSI methods can result in fertilization *in vitro* and the development potential of the corresponding embryos was similar (Table 2).

### Ratio of uterine endometrial to myometrial thicknesses is crucial for embryo implantation

Implantation depends on synchronization between embryo development and receptive endometrium^22^. Many factors could affect the endometrial receptivity of recipients^23^,^24^. To further clarify the factors that heavily affect embryo implantation, we carried out the following experiment. First, we transferred one-cell embryos from one batch of fertilized oocytes from the same oocyte and sperm donors into two or three recipients. After a month, pregnancy diagnosis was made. Then, a pregnant and a non-pregnant monkeys that received the same batch embryos were chosen as a trial pair (Table 3), and in this way we obtained 15 pairs of cynomolgus monkeys. We analyzed their B-mode ultrasound image data in uterus and ovaries captured just before embryo transfer (Supplementary Fig. S6A–D). Interestingly, we found that the ratio (3.2 ± 0.3) of endometrial to myometrial thicknesses in abdominal ultrasonic transverse section of uterus in the pregnancy group was much higher than that (2.0 ± 0.1) in the non-pregnant group (P < 0.01) (Table 3). We also retrospectively analyzed data from 10 pairs of rhesus monkeys treated with the same experiment protocol and obtained similar results (Supplementary Table S1). On the other hand, the sizes of the uterus and ovaries, serum E2/P4 levels (rhesus monkeys), day of menstrual cycles at embryo transfer, length of menstrual cycles just before transfer, and either dominant follicles or freshly-ovulated follicles at transfer, all showed no differences between the pregnancy and non-pregnancy groups (Table 3 and Supplementary Table S1). The above results clearly indicated that, the ratio of endometrial to myometrial thicknesses in abdominal ultrasonic transverse section of uterus at embryo transfer is a potential indicator for selecting recipients when they are around the time of ovulation with normal menstrual cycles in both cynomolgus and rhesus monkeys.

### Cesarean section for cynomolgus baby delivery

Not surprisingly, abortion rate was higher in triplet (50%) pregnancy compared with twin (25%) and single (25%) pregnancies in cynomolgus monkeys (Table 4), although no statistical analysis was conducted due to small sample sizes. In natural delivery, gestation periods were 161.9, 148.4 and 142.5 days, and infant survival rates were 86.7% (13/15), 78.6% (11/14) and 33.3% (2/6), respectively for pregnancy with single, twin and triplet fetuses. It was clear that gestation period shortened and infant survival rate decreased with the increase of fetus numbers in pregnancy. Therefore, more attention should be paid for maintaining multiple fetus pregnancy and managing their delivery in cynomolgus monkeys need to be improved. In present study, we carried out six cesarean deliveries and the survival rate of infant monkeys was 100% for single, twin and triplet pregnancies (Table 4). Our results suggest that infant survival rates can be improved by cesarean delivery in multiple fetus pregnancy in cynomolgus monkeys.

## Discussion

**Figure 3:**
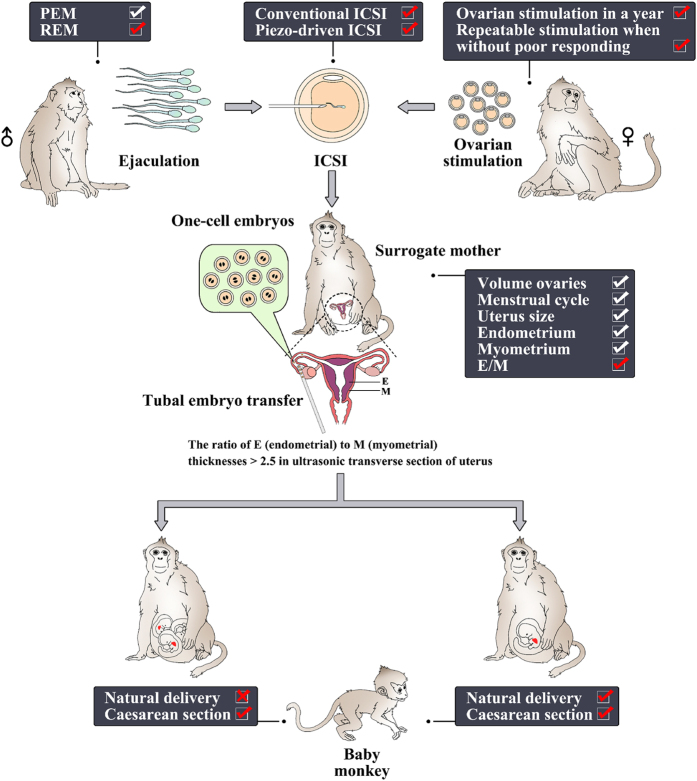
The highlights in ARTs in cynomolgus monkeys investigated in the present study. Optimize the screening criterions for selecting sperm, oocyte, and embryo as well as its surrogate mothers that enhance efficiency of assisted reproduction technologies in cynomolgus monkeys. REM, rectal probe electrical stimulation method; PEM, penile probe electrical stimulation; ICSI, intracytoplasmic sperm injection.

In the present study, we investigated some of the current problems of ARTs in cynomolgus monkeys and demonstrated successful solutions. Our key findings are: the thickness ratio of uterine endometrium to myometrium may be a reliable indicator for selecting embryo recipients; ovarian stimulation can be done year-round and repeated for six times in a monkey without dramatic decrease in oocyte number and quality, until a poor response happened; fertilization can be readily achieved with conventional as well as piezo-driven intracytoplasmic sperm injection procedures; in comparison of semen collection methods, seminal weight and sperm quality on the basis of motility, malformation and acrosomal integrity of sperm are higher in PEM than that in REM; moreover, cesarean section might be an effective strategy for increase baby survival rates of multiple pregnancy (Fig. 3).

Electro-stimulation ejaculation is commonly adopted to collect semen for reproductive research in nonhuman primates^25^,^26^,^27^. To our knowledge, the present study is the first report on comprehensive comparison of REM and PEM in cynomolgus monkeys. Our study indicates that, in general, REM is simple and fast while PEM provides semen of high quality. But we found that urea concentration in REM semen is much higher than that in PEM semen. There was a significant negative relationship between sperm motility and urea concentration^16^, which could explain the low sperm motility in REM semen and high motility in PEM semen. A urination reflex could be likely triggered during electrical stimulation since the rectal probe was proximity to the spine. In addition, male monkeys showed some signs of distress during REM process, whereas animals stayed relatively quiet and comfortable during PEM process. Combined with the high quality of semen and sperm from PEM, we suggest that PEM should be used as primary ejaculation method for both scientific and animal welfare reasons.

In rhesus monkeys, we and some researchers reported that age, body weight, season, the day of the menstrual cycle for starting ovarian stimulation, ovarian stimulation regimen, and repetition stimulation, all affected the outcome of ovarian stimulation^12^,^28^,^29^,^30^,^31^,^32^,^33^. In present study, our findings demonstrated that cynomolgus monkeys are appropriate for ovarian stimulation throughout the year and that ovarian recovery is complete within two menstrual cycles after ovarian stimulation, which is consistent with a previous report in rhesus monkeys^18^. Previous studies reported that ovaries of tammar wallabies^34^ and rhesus monkeys^35^ responded well to consecutive stimulations if the same ovarian stimulation protocol was repeat. In present study, we found that the number of oocytes recovered per stimulation cycle did not significantly change until the sixth stimulations in responding animals; this finding was also consistent with a previous report in rhesus monkeys^35^ and mice^36^. Here, we highlight that animals with good response to ovarian stimulation generally respond well to subsequent ovarian stimulations. Once animals respond poorly to an ovarian stimulation, they remain to respond poorly to subsequent stimulations. Hence, once an animal responds poorly to certain ovarian stimulation, it should not be enrolled in ovarian stimulation any more. Therefore, the present findings will be helpful for improving the efficiency of ovarian stimulation in monkeys. Moreover, combined with our previous study^19^ that the number of small follicles (>3 mm) in both ovaries at the beginning of ovarian stimulation, our findings thus can provide a prediction with relative accuracy for the results of ovarian stimulation.

It was suggested that consecutive ovarian stimulation could impair ovarian responses^37^. Repeated ovarian stimulation may induce the production of oocytes with low developmental competence due to oxidative damage and mitochondrial DNA mutations^36^. Some studies indicated that repetitive ovarian stimulations affected oocyte spindle morphology but did not induce changes in a set of proteins involved in cell cycle progression^38^. For such oocytes, *in vivo* compensatory mechanisms that optimize the developmental competence may be recruited, as in mice^39^. Nevertheless, a consensus has not been established regarding the adverse effect of repeated ovarian stimulations. In the present study, we showed that the fertilization rate of mature oocytes did not differ among six consecutive repeated stimulations in cynomolgus monkeys. Since bio-assay of quality standard for oocytes has not been established in mammals, the full-term developmental potential of oocytes retrieved after consecutive ovarian stimulations should be further studied in detail in the future. ARTs, comprising ovarian stimulation and oocyte pick-up, aim at high rates of blastocysts formation and survival of the transferred embryos to term. Beyond this, all treatments applied should be safe for the donor animal, and without causing any compromise to its welfare and to its future fertility. To address the possibility that treatment of cynomolgus monkeys with gonadotropins might affect their future reproductive performance, after oocyte pick-up, cynomolgus monkeys were mated with fertile males and pregnancy diagnoses were performed per month by B-mode ultrasound scanner. We did not observe significant alteration of the capability of pregnancy female monkeys. Indeed, we detected higher pregnant ratio for these female monkeys (3 of 7 female monkeys in our experimental group were pregnant when paired with a male monkey together in a month, while 86 of 305 female monkeys developed into pregnancy in untreated controlled group. Clearly, the rate of pregnancy of female monkeys stimulated is acceptable as compared to controls, which was also consisted with previous reported in rhesus monkeys^35^,^40^.

Both endometrium and myometrium undergo significant changes during reproductive cycle^41^,^42^,^43^, controlled by ovarian estrogen and progesterone^44^. Receptive endometrium is essential for embryo implantation^45^,^46^. Some studies in human reported that endometrial thickness greater than or equal to10 mm was most desirable for embryo implantation^47^, while others claimed that thickness less than 4 mm was optimal^48^. Surprisingly, some else studies showed that endometrial thickness had no relationship with pregnancy^49^. Thus, there seems to be some controversies about endometrial thickness as a factor in embryo implantation. In present study, the ratio of endometrial to myometrial thicknesses in abdominal ultrasonic transverse section of uterus was very high in the pregnancy group in both cynomolgus and rhesus monkeys. Moreover, we have investigated implantations of eight surrogate mothers who received several embryos at various developmental stages ranging from 8-cell stage to blastocyst, followed by recording the ultrasound images of uterus at embryo transfer, we found that all the female monkeys develop pregnancy shown the ratio of endometrial to myometrial thicknesses more than three in abdominal ultrasonic transverse section of uterus (data not shown). Therefore, we suggest that the thickness ratio greater than 2.5 in abdominal ultrasonic transverse section of uterus could be adopted as a selection index for embryo transfer recipients in monkeys. Our finding could markedly improve embryo transfer efficiency in primates. Maintaining multiple pregnancies is difficult in cynomolgus monkeys, probably due to the limitation of reproductive system that generally give birth to only one fetus. Multiple births must incur a heavy load for their mothers, even before, and, of course, after delivery in terms of sucking and carrying two infants during traveling. In our present study, cesarean section not only decrease the dystocia rate, but also improve the survival rate of infant and pregnant mother.

Gene editing with the recent developed ZFN, TALNE and CRISPR/Cas toolboxs have made the genetic manipulation of monkeys possible. As ARTs and gene editing technologies are indispensable compositions for generation of genetically modified monkeys. Less efficiency of ARTs of monkeys absolutely causes low efficiency of creation of genetically-modified monkeys. So ARTs are very important for generation of genetically-modified monkeys. Although, in this manuscript, we did not incorporate any gene editing data in monkeys, we did spend quite a lot of efforts in generating disease monkey model by the cutting edge TALEN and CRISPR/Cas tools^8^.

Taken together, here we show that compared to REM, PEM produces higher semen volume and better semen quality on the basis of motility, malformation and acrosomal integrity of sperm. Ovarian stimulation can be performed in all seasons, and recovery of oocytes and their fertilization ability remain fairly steady throughout the year. Moreover, cynomolgus monkeys can respond to repeated ovarian stimulation up six times separated at intervals of 2–4 menstrual cycles before the response to stimulation becomes poor. Both conventional and piezo-driven ICSI procedures can be used for fertilization *in vitro* without difference in embryo development rate. We find that the ratio of endometrial to myometrial thicknesses in abdominal ultrasonic transverse section of uterus is the most reliable indicator for selection of recipients for embryo transfer. Furthermore, we find that caesarean section is an effective strategy for increasing baby survival rates for twin and multiple births resulted from embryo transfer. The findings provides extensive and detailed data for reproductive biology in nonhuman primates, which will be useful not only for enhancing ARTs efficiency in macaque monkeys, but also for facilitating their use in generation of genetically-modified monkeys for biomedical research.

## Materials and Methods

### Monkeys and chemicals

All cynomolgus monkeys were provided and raised by Blooming-Spring Biotechnology Development Co., Ltd., of Guangdong (BBDC), which is an accredited member of the Association for Assessment and Accreditation of Laboratory Animal Care. Healthy, sexually-mature male monkeys (age 6–11 years, body weight 6.5–13 kg) and female monkeys (age 4.5–9 years, body weight 3.5–5 kg) were involved in the present study. All animal procedures such as semen collection, ovarian stimulation, oocyte recovery, abdominal ultrasonography, embryo transfer and cesarean section were approved by the Institutional Animal Care and Use Committee of BBDC and were carried out in accordance with the Guide for the Care and Use of Laboratory Animals. Unless stated otherwise, all chemicals were obtained from Sigma Chemical Co. (St. Louis, MO, USA).

### Semen collection

REM was conducted as previously described^50^ with some improvements. The voltage output of the stimulator (Lane Manufacturing Inc., USA) was controlled by a regulator switch with 9 steps. Monkeys were anesthetized with ketamine hydrochloride (Lianyungang International Trade Co., Ltd., Lianyungang City, China) at dose of 10–12 mg/kg body weight injected intramuscularly. They were held in a supine position on an operating table with four limbs tied. Penis was cleaned with warmed saline on absorbent cotton. A lubricated probe was inserted into rectum for 3–4 cm and positioned with both electrodes oriented in mid-ventral direction. Each electrical stimulation consisted of pulses of 4–6 s duration with 2–3 s rest in between. Stimulation started at step 1 for five repeats. If no painful response was observed, stimulation was continued with the voltage raised to next step. Thus, step by step, stimulation was repeated and intensified until ejaculation occurred. If ejaculation did not occur after stimulation at step 9, the animal was rested for 10 min, and the stimulation protocol repeated from step 1 again. If ejaculation still did not occur, no further attempt to ejaculation was made.

PEM was conducted as previously described^51^ with some improvements. A multi-purpose stimulator (C4V2a, Shanghai Jialong Teaching Instrument Factory, Shanghai, China) was used. The limbs of anesthetized animals were tied to the operating table. After the penis was drawn out of the prepuce and washed with warmed saline, a circular electrode made of copper wire (wrapped in absorbent cotton with warmed saline) was applied to the base of the penile shaft. Another circular electrode was wrapped around the base of the glans penis. Stimulation bandwidth of 10 ms and frequency of 25 Hz was set as constant. Voltage output was stepped at 8 V interval from 8 V to 56 V. Electrical stimulations were maintained for 10 s with 2–3 min rest and stimulations were repeated twice at each step. Electrical stimulations started at 8 V. If ejaculation did not occur, stimulations continued with the voltage raised to next step. Thus, step by step, stimulations were repeated and intensified until ejaculation occurred. If ejaculation did not occur after the stimulation intensity at 56 V, the animal was rested for 10 min, and the stimulation protocol repeated to start at 8 V again. If ejaculation still did not occur yet, no further attempt to ejaculation was made.

Ejaculates were collected directly from the tip of the penis after ejaculation into warmed tubes. Prior to measuring the characteristics of semen, ejaculates were kept in water bath at 37 °C^52^.

### Measurements of pH, weight and sperm density, motility, malformations and acrosomal integrity in semen

pH, weight, and sperm density in semen were measured with reference to international standards^53^. The percentage of motile sperm was assessed under a light microscope at 37 °C^54^. Briefly, a 10-μL drop of semen sample was deposited onto a pre-warmed Neubauer hemacytometer (BOECO, Hamburg, Germany) and covered with a coverslip. The slide was incubated on a 37 °C warming plate for 2 min, and then the motility of sperm was evaluated by counting approximately 200 sperm. An experienced evaluator, who examined all the specimens, assessed motility. The sperm malformation rate was evaluated by counting sperm microscopically after staining with diff-quick sperm dyeing liquid (Diff-quick kit, Senbeijia, Nanjing, China)^53^. Those sperm with neck, midpiece or principal piece defects, even those with only a head, were recorded as malformed^53^. Alexa Fluor-488-PNA (peanut agglutinin) conjugate staining (Molecular Probes, Eugene, OR, USA) was used to determine sperm acrosome integrity^55^. Sperm with intact acrosomes showed uniform apple-green fluorescence in the acrosomal region of the sperm head (acrosomal cap), while sperm displaying only partially green fluorescence of the acrosomal cap, indicating its breakdown (partially damaged acrosome), and sperm displaying no fluorescence, indicating a complete loss of the outer acrosomal membrane (damaged acrosome), were recorded as lacking acrosomal integrity.

### Measurement and addition of urea in semen

Urea in seminal plasma was measured with a biochemical analyzer (VetTestTM, IDEXX, USA) following the manufacturer’s instructions with some improvements. Accuracy testing was done four times with serum of male cynomolgus monkeys and recovery of urea added into the serum was more than 90% in actual concentrations of 8 mmol/L, 11 mmol/L, 14 mmol/L and 18 mmol/L (Supplementary Fig. S2A). Urea concentrations in seminal plasma freed of sperm by centrifugation were measured and then required concentrations of urea in seminal plasma were made by adding 500 mM urea saline.

### Vaginal bleeding monitoring

Female cynomolgus monkeys were monitored twice a day (8:00 and 20:00) for vaginal bleeding to detect menstruation onset. Menstrual cycle period was defined as the duration from the first day of vaginal bleeding to the day before next vaginal bleeding.

### Ovarian stimulation and oocyte recovery

As shown in Supplementary Fig. S3, administrations of rhFSH and rhCG (Gonal F, Laboratories Serono SA, Aubonne, Switzerland) were performed as previously described^18^,^32^. Cumulus-oocyte complexes were collected by laparoscopic follicular aspiration and oocytes were stripped of cumulus cells by pipetting after brief exposure (<1 min) to hyaluronidase (0.5 mg/mL) at 37 °C. Oocytes were classified as metaphase II (MII; with first polar body), metaphase I (MI; no germinal vesicle, no first polar body), or prophase I (PI or GV, intact germinal vesicle). The numbers of MI, MII and GV oocytes were counted for statistical analysis. Total oocytes included oocytes at MI, MII and GV stages in a stimulation trial. Oocytes were then collected for fertilization with ICSI and the resulting zygotes were used for either embryo transfer or other studies. Good responders or poor responders are defined as our previously study^32^. In which, a good responder is referred to a monkey that can provide more than five MI and MII oocytes in one ovarian stimulation. While, a poor responder is defined for those monkeys who can only provide not more than five MI and MII oocytes in one ovarian stimulation procedure. Rate of responding animals meant the good responders to all animals stimulated in a trial.

### Intracytoplasmic sperm injection and embryo culture

The conventional ICSI procedure was performed with commercially prepared injection and holding pipettes and an Eppendorf micromanipulation system attached to an Olympus IX 70 inverted microscope with Hoffman Contrast optics, as previously described^20^ and the piezo-driven ICSI procedure was performed with a piezo-micropipette-driving unit (Prime Tech, City, Japan), as previously described^56^. After ICSI, the constituted oocytes were transferred into 50-μL drop of HECM-9 medium balanced in advance at 37 °C in a humidified 5% CO_2_ in air under the mineral oil, 10–12 h later oocytes exhibiting two pronuclei were considered as fertilization. The fertilization rate meant the fertilized oocytes to all constituted oocytes in a trial.

### Abdominal ultrasonography

Uterus and ovaries of recipients were examined with a real time B-mode ultrasound scanner (15–38 MHz transducers, VEVO 2100E, Visualsonics Co., Ltd., Canada) on the day of and just before embryo transfer, and 30 days after embryo transfer. Female monkeys were bound in a supine position under anesthesia on an operating table with the help of the manipulator’s finger inserted into the rectum to control the position of the uterus horizontally under the transducer. The transducer was placed on lower midline abdomen of the animal to locate the uterus in a transverse plane. The transducer was then moved slowly and laterally to obtain the transverse section of the uterine body and all images were recorded. According to the reflectivity of the uterus, the images were divided into five grades^57^. The first grade images were selected to distinguish the outline of the uterus, endometrium and myometrium. The uterine body and endometrium were observed precisely by dense-echo from the edge of the uterus and endometrium. The length of the uterus and endometrium was measured on the maximal horizontal axis and the height of the uterus and the thicknesses of endometrium and myometrium on the maximal longitudinal axis. Endometrial (showed slight low ultrasonic echo) thickness was the distance between the up interface (represented the endometrium-myometrial junction) and the down interface (represented the contralateral junction) in the maximal abdominal ultrasonic transverse section of uterus. Myometrial thickness was the height of the uterus minus the endometrial thickness and then divided by two. Both ovaries were scanned independently and the measurements were made with omnidirectional calipers superimposed on static or frozen ultrasound images on the screen. E/M meant ratio of endometrial (E) to myometrial (M) thicknesses. All examinations were performed by one person and all ultrasound images were originals. In addition, dominant follicle represented round follicle with diameter at 3 to 6 mm and transparent thin wall, and freshly ovulated follicle meant new stigma (corpus hemorrhagicum) or new corpus luteum on ovary shown as irregular follicle in ultrasonic images, both of which were also checked under laparoscopy at embryo transfer. Animals were diagnosed as pregnancy by imaging an apparent conceptus (anechoic, elongated structure) with beating heart.

### Embryo transfer

Multiparous females with two consecutive normal menstrual cycles (26–32 days) and with normal ovaries containing dominant follicle or freshly ovulated follicle, and uterus with the normal echo in uterus cavity checked by ultrasound immediately just before embryo transfer were selected as embryo recipients. We drew blood of rhesus monkeys respectively and used rapid radioimmunoassays kit (Tianjin Jiuding Medical and Biological Engineering Co., Ltd., Tianjin, China) to measure serum E2 and P4 levels. One-cell embryos were transferred into one oviduct of each recipient via laparoscopy with a fixed polythene catheter (outside and inside diameters of 1.09 and 0.38 mm, respectively), threaded through a 25-gauge hypodermic needle^32^.

### Cesarean section

Approaching parturition (140–160 days), pregnant monkeys showed either anxious, week of moving and loss of appetite, or appearance of blood and amniotic fluid from vagina, then they were anesthetized and cesarean section and postoperative care were carried out as per routine protocol^58^. Newborns were kept in a premature baby incubator at 35 °C. Infants were fed artificially with preemy milk 6 times a day and food intake was increased progressively with infant body weight gain.

## Additional Information

**How to cite this article**: Ma, Y. *et al*. Efficient production of cynomolgus monkeys with a toolbox of enhanced assisted reproductive technologies. *Sci. Rep*. **6**, 25888; doi: 10.1038/srep25888 (2016).

## Supplementary Material

Supplementary Information

## Figures and Tables

**Table 1:** Success Rate of Ejaculation, Time-Consumed, and Weight, pH and Density of Semen in REM and PEM.

Stimulus method	Number of stimulations	First ejaculation (%)	Stimulus time to the first ejaculation (min)	All ejaculation (%)	Stimulations time to all ejaculation (min)	Semen weight (g)	Semen pH	Sperm concentration (10^9^/mL)
REM	32	8 (100.0)	8.13 ± 0.60^a^	32 (100.0)	9.13 ± 0.91^a^	0.14 ± 0.03^a^	7.36 ± 0.13	1.14 ± 0.35^b^
PEM	32	6 (75.0)	11.00 ± 0.45^a^	27 (84.4)	16.62 ± 1.03^a^	0.28 ± 0.04^a^	7.13 ± 0.11	2.89 ± 0.29^b^

Superscripts ‘a’ within a column denote differences between groups: *P* < 0.05. Superscripts ‘b’ within a column denote significant differences between groups: *P* < 0.01.

**Table 2:** Fertilization of Cynomolgus Oocytes by Conventional and Piezo-Driven ICSI and Subsequent Embryo Development.

ICSI method	Oocytes	Oocytes surviving after injection (%)	Fertilized oocytes with two pronuclei (%)	Developing embryos		
				2-cell	8-cell	Blastocyst (%)
Piezo-driven	78	75 (96.2)	61 (81.3)	59	55	24 (40.7)
Conventional	101	95 (94.1)	67 (70.5)	59	59	33 (55.9)

**Table 3:** Parameters of Uterus and Ovaries at Embryo Transfer of Pregnant and Non-Pregnant Cynomolgus Recipients Received Embryos from the Same Fertilization Trial.

Parameter	Pregnant monkeys just before ET			Non-pregnant monkeys just before ET		
	Max	Min	Means ± s.e.m.	Max	Max	Means ± s.e.m.
Body weight (kg)	4.8	3.5	4.1 ± 0.1	4.9	3.1	4.2 ± 0.1
Age (year)	10.0	7.0	8.2 ± 0.2	10.0	6.0	8.1 ± 0.2
Volume ovary (cm^3^)	13.5	1.9	8.6 ± 1.5	11.9	4.4	8.4 ± 1.0
Menstrual cycle (day)	39	21	2 8 ± 5.3	36	24	3 0 ± 3.6
Duration of MC just before embryo transfer (day)	16.0	10.0	11.7 ± 0.4	16.0	9.0	11.4 ± 1.2
Dominant follicle/Freshly ovulated follicle	13/5			8/10		
Embryos transferred	8.0	4.0	6.7 ± 0.5	8.0	3.0	5.8 ± 0.7
Uterus length (mm)	26.4	15.7	22.1 ± 0.7	29.7	15.3	20.3 ± 0.8
Endometrium length (mm)	20.4	8.3	13.8 ± 0.8	16.3	7.3	12.0 ± 0.7
Uterus height (mm)	18.8	12.3	15.7 ± 0.5	22.3	11.2	16.0 ± 0.8
Endometrium thickness (mm)	13.0	7.1	9.2 ± 0.4	12.3	5.2	7.9 ± 0.4
Myometrium thickness (mm)	4.4	1.2	3.2 ± 0.3	5.6	2.7	3.9 ± 0.2
E/M	4.0	2.5	3.2 ± 0.3	2.5	1.1	2.0 ± 0.1**

Data are means ± s.e.m. with n = 15 per group. P-values were analyzed with Student’s t-test (nonparametric test, two tailed), **P < 0.01. Expect dominant follicle/freshly ovulated follicle with Fisher’s exact test. Max, maximum value; Min, minimum value; ET, embryo transfer; E2, estradiol; P4, progesterone; E/M = endometrial to myometrial thicknesses.

**Table 4:** Cynomolgus Infant Survival in Natural Birth and Cesarean Section.

Pregnancy	Case	Abortion			Natural birth			Cesarean section		
		Case	Duration of pregnancy (day)	Abortion rate (%)	Case	Gestation period (day)	Infant survival (%)	Case	Duration of pregnancy (day)	Infant survival (%)
Singleton	24	6	99.0 ± 11.4	6/24 (25.0)	15	161.9 ± 2.2	13 (86.7)	3	152.7 ± 6.2	3 (100.0)
Twins	12	3	106.0 ± 25.2	3/12 (25.0)	7	148.4 ± 2.7	11 (78.6)	2	152.0 ± 2.0	4 (100.0)
Triplets	6	3	115.3 ± 13.7	3/6 (50.0)	2	142.5 ± 2.5	2 (33.3)	1	154.0	3 (100.0)

## References

[b1] Thomas K. R. & Capecchi M. R. Site-directed mutagenesis by gene targeting in mouse embryo-derived stem cells. Cell 51, 503–512 (1987). 2822260 10.1016/0092-8674(87)90646-5

[b2] Geurts A. M. . Knockout rats via embryo microinjection of zinc-finger nucleases. Science 325, 433 (2009). 19628861 10.1126/science.1172447 PMC2831805

[b3] Anguela X. M. . Robust ZFN-mediated genome editing in adult hemophilic mice. Blood 122, 3283–3287 (2013). 24085764 10.1182/blood-2013-04-497354 PMC3821724

[b4] Carlson D. F. . Efficient TALEN-mediated gene knockout in livestock. Proc Natl Acad Sci USA 109, 17382–17387 (2012). 23027955 10.1073/pnas.1211446109 PMC3491456

[b5] Li W., Teng F., Li T. & Zhou Q. Simultaneous generation and germline transmission of multiple gene mutations in rat using CRISPR-Cas systems. Nat Biotechnol 31, 684–686 (2013). 23929337 10.1038/nbt.2652

[b6] Wang X. . Efficient CRISPR/Cas9-mediated biallelic gene disruption and site-specific knockin after rapid selection of highly active sgRNAs in pigs. Sci Rep 5, 13348 (2015). 26293209 10.1038/srep13348 PMC4543986

[b7] Niu Y. . Generation of gene-modified cynomolgus monkey via Cas9/RNA-mediated gene targeting in one-cell embryos. Cell 156, 836–843 (2014). 24486104 10.1016/j.cell.2014.01.027

[b8] Wan H. . One-step generation of p53 gene biallelic mutant Cynomolgus monkey via the CRISPR/Cas system. Cell Res 25, 258–261 (2015). 25430965 10.1038/cr.2014.158 PMC4650568

[b9] Liu H. . TALEN-mediated gene mutagenesis in rhesus and cynomolgus monkeys. Cell Stem Cell 14, 323–328 (2014). 24529597 10.1016/j.stem.2014.01.018 PMC4024384

[b10] Liu Z. . Autism-like behaviours and germline transmission in transgenic monkeys overexpressing MeCP2. Nature 530, 98–102 (2016). 26808898 10.1038/nature16533

[b11] Bavister B. D., Boatman D. E., Collins K., Dierschke D. J. & Eisele S. G. Birth of rhesus monkey infant after *in vitro* fertilization and nonsurgical embryo transfer. Proc Natl Acad Sci USA 81, 2218–2222 (1984). 6326113 10.1073/pnas.81.7.2218 PMC345469

[b12] Ma Y. & Yang S. Advances in Genetically Modified Chinese Macaques. Progress in Biochemistry and Biophysics 41, 1089–1098 (2014).

[b13] Sun Q. . Efficient reproduction of cynomolgus monkey using pronuclear embryo transfer technique. Proc Natl Acad Sci USA 105, 12956–12960 (2008). 18725640 10.1073/pnas.0805639105 PMC2529107

[b14] Sasaki E. . Generation of transgenic non-human primates with germline transmission. Nature 459, 523–527 (2009). 19478777 10.1038/nature08090

[b15] Shimozawa N., Nakamura S., Takahashi I., Hatori M. & Sankai T. Characterization of a novel embryonic stem cell line from an ICSI-derived blastocyst in the African green monkey. Reproduction 139, 565–573 (2010). 19955206 10.1530/REP-09-0067

[b16] Bozkurt Y. Relationships between seminal plasma composition and sperm quality parameters of the Salmo trutta macrostigma (Dumeril, 1858) semen: with emphasis on sperm motility. Czech J. Anim. Sci 56, 9 (2011).

[b17] Timmermans P. J., Schouten W. G. & Krijnen J. C. Reproduction of cynomolgus monkeys (Macaca fascicularis) in harems. Lab Anim 15, 119–123 (1981). 7278111 10.1258/002367781780958991

[b18] Yang S. . Effects of rhFSH regimen and time interval on ovarian responses to repeated stimulation cycles in rhesus monkeys during a physiologic breeding season. Theriogenology 70, 108–114 (2008). 18456315 10.1016/j.theriogenology.2008.03.012

[b19] Yang S. . Dynamic changes in ovarian follicles measured by ultrasonography during gonadotropin stimulation in rhesus monkeys. Theriogenology 72, 560–565 (2009). 19501391 10.1016/j.theriogenology.2009.04.012

[b20] Ogonuki N., Sankai T., Cho F., Sato K. & Yoshikawa Y. Comparison of two methods of assisted fertilization in cynomolgus monkeys (Macaca fascicularis): intracytoplasmic sperm injection and partial zona dissection followed by insemination. Hum Reprod 13, 2555–2560 (1998). 9806282 10.1093/humrep/13.9.2555

[b21] Nusser K. D. . Developmental competence of oocytes after ICSI in the rhesus monkey. Hum Reprod 16, 130–137 (2001). 11139551 10.1093/humrep/16.1.130

[b22] Turnbull L. W., Lesny P. & Killick S. R. Assessment of uterine receptivity prior to embryo transfer: a review of currently available imaging modalities. Hum Reprod Update 1, 505–514 (1995). 9080224 10.1093/humupd/1.5.505

[b23] Lutjen P. . The establishment and maintenance of pregnancy using *in vitro* fertilization and embryo donation in a patient with primary ovarian failure. Nature 307, 174–175 (1984). 6690997 10.1038/307174a0

[b24] Wright L. J. . Progesterone monoclonal antibody blocks pregnancy in mice. Nature 295, 415–417 (1982). 7057905 10.1038/295415a0

[b25] Gould K. G., Warner H. & Martin D. E. Rectal probe electroejaculation of primates. J Med Primatol 7, 213–222 (1978). 105137 10.1159/000459881

[b26] Gould K. G. & Mann D. R. Comparison of electrostimulation methods for semen recovery in the rhesus monkey (Macaca mulatta). J Med Primatol 17, 95–103 (1988). 3418685

[b27] Matsubayashi K. Comparison of the two methods of electroejaculation in the Japanese monkey (Macaca fuscata). Jikken Dobutsu 31, 1–6 (1982). 7075683 10.1538/expanim1978.31.1_1

[b28] Schramm R. D. & Bavister B. D. Follicle-stimulating hormone priming of rhesus monkeys enhances meiotic and developmental competence of oocytes matured *in vitro*. Biol Reprod 51, 904–912 (1994). 7849192 10.1095/biolreprod51.5.904

[b29] Abbasi R., Kenigsberg D., Danforth D., Falk R. J. & Hodgen G. D. Cumulative ovulation rate in human menopausal/human chorionic gonadotropin-treated monkeys: “step-up” versus “step-down” dose regimens. Fertil Steril 47, 1019–1024 (1987). 3109955

[b30] Wolf D. P. . *In vitro* fertilization and embryo transfer in the rhesus monkey. Biol Reprod 41, 335–346 (1989). 2508776 10.1095/biolreprod41.2.335

[b31] Wolf D. P., Thomson J. A., Zelinski-Wooten M. B. & Stouffer R. L. *In vitro* fertilization-embryo transfer in nonhuman primates: the technique and its applications. Mol Reprod Dev 27, 261–280 (1990). 2078341 10.1002/mrd.1080270313

[b32] Yang S. . Effects of rhFSH dose on ovarian follicular response, oocyte recovery and embryo development in rhesus monkeys. Theriogenology 67, 1194–1201 (2007). 17321585 10.1016/j.theriogenology.2006.10.021

[b33] Yang S., He X., Hildebrandt T. B., Zhou Q. & Ji W. Superovulatory response to a low dose single-daily treatment of rhFSH dissolved in polyvinylpyrrolidone in rhesus monkeys. Am J Primatol 69, 1278–1284 (2007). 17440965 10.1002/ajp.20433

[b34] Magarey G. M., Rodger J. C., Buist J. M. & Mate K. E. Effects of repeated superovulation and surgical oocyte collection on ovarian response and natural breeding ability of the tammar wallaby (Macropus eugenii). Reproduction 125, 701–707 (2003). 12713433 10.1530/rep.0.1250701

[b35] VandeVoort C. A. & Tarantal A. F. Recombinant human gonadotropins for macaque superovulation: repeated stimulations and post-treatment pregnancies. J Med Primatol 30, 304–307 (2001). 11990529 10.1034/j.1600-0684.2001.300603.x

[b36] Chao H. T. . Repeated ovarian stimulations induce oxidative damage and mitochondrial DNA mutations in mouse ovaries. Ann N Y Acad Sci 1042, 148–156 (2005). 15965057 10.1196/annals.1338.016

[b37] Diamond M. P., DeCherney A. H., Hill G. A., Nero F. & Wentz A. C. Response to repetitive cycles of ovulation induction in the same women. J In Vitro Fert Embryo Transf 4, 251–255 (1987). 10.1007/BF01555198 3694005

[b38] Di Luigi G. . Repeated ovarian stimulation does not affect the expression level of proteins involved in cell cycle control in mouse ovaries and fallopian tubes. J Assist Reprod Genet 31, 717–724 (2014). 24619509 10.1007/s10815-014-0198-z PMC4048378

[b39] Combelles C. M. & Albertini D. F. Assessment of oocyte quality following repeated gonadotropin stimulation in the mouse. Biol Reprod 68, 812–821 (2003). 12604630 10.1095/biolreprod.102.008656

[b40] Yang S. . Ovarian response to gonadotropin stimulation in juvenile rhesus monkeys. Theriogenology 72, 243–250 (2009). 19362733 10.1016/j.theriogenology.2009.02.019

[b41] Burroughs K. D., Fuchs-Young R., Davis B. & Walker C. L. Altered hormonal responsiveness of proliferation and apoptosis during myometrial maturation and the development of uterine leiomyomas in the rat. Biol Reprod 63, 1322–1330 (2000). 11058535 10.1095/biolreprod63.5.1322

[b42] Shynlova O. . Myometrial apoptosis: activation of the caspase cascade in the pregnant rat myometrium at midgestation. Biol Reprod 74, 839–849 (2006). 16407500 10.1095/biolreprod.105.048124

[b43] Minas V., Loutradis D. & Makrigiannakis A. Factors controlling blastocyst implantation. Reprod Biomed Online 10, 205–216 (2005). 15823225 10.1016/s1472-6483(10)60942-x

[b44] Cha J., Sun X. & Dey S. K. Mechanisms of implantation: strategies for successful pregnancy. Nat Med 18, 1754–1767 (2012). 23223073 10.1038/nm.3012 PMC6322836

[b45] Revel A. Defective endometrial receptivity. Fertil Steril 97, 1028–1032 (2012). 22542142 10.1016/j.fertnstert.2012.03.039

[b46] Yoshinaga K. Uterine receptivity for blastocyst implantation. Ann N Y Acad Sci 541, 424–431 (1988). 3057996 10.1111/j.1749-6632.1988.tb22279.x

[b47] Isaacs J. D. Jr. . Endometrial thickness is a valid monitoring parameter in cycles of ovulation induction with menotropins alone. Fertil Steril 65, 262–266 (1996). 8566245 10.1016/s0015-0282(16)58082-0

[b48] Sundstrom P. Establishment of a successful pregnancy following *in-vitro* fertilization with an endometrial thickness of no more than 4 mm. Hum Reprod 13, 1550–1552 (1998). 9688390 10.1093/humrep/13.6.1550

[b49] Ng E. H., Chan C. C., Tang O. S., Yeung W. S. & Ho P. C. Factors affecting endometrial and subendometrial blood flow measured by three-dimensional power Doppler ultrasound during IVF treatment. Hum Reprod 21, 1062–1069 (2006). 16373406 10.1093/humrep/dei442

[b50] Weisbroth S. & Young F. A. The Collection of Primate Semen by Electro-Ejaculation. Fertil Steril 16, 229–235 (1965). 14261227 10.1016/s0015-0282(16)35530-3

[b51] Ramesh V., Ramachandra S. G., Krishnamurthy H. N. & Rao A. J. Electroejaculation and seminal parameters in bonnet monkeys (Macaca radiata). Andrologia 30, 97–100 (1998). 9629430 10.1111/j.1439-0272.1998.tb01153.x

[b52] Settlage D. S. & Hendrickx A. G. Electroejaculation technique in Macaca mulatta (rhesus monkeys). Fertil Steril 25, 157–159 (1974). 4204296

[b53] Lu J. C., Huang Y. F. & Lu N. Q. [WHO Laboratory Manual for the Examination and Processing of Human Semen: its applicability to andrology laboratories in China]. Zhonghua Nan Ke Xue 16, 867–871 (2010). 21243747

[b54] Saragusty J., Gacitua H., Rozenboim I. & Arav A. Protective effects of iodixanol during bovine sperm cryopreservation. Theriogenology 71, 1425–1432 (2009). 19299004 10.1016/j.theriogenology.2009.01.019

[b55] Cooper T. G. & Yeung C. H. A flow cytometric technique using peanut agglutinin for evaluating acrosomal loss from human spermatozoa. J Androl 19, 542–550 (1998). 9796613

[b56] Ogonuki N. . Pregnancy by the tubal transfer of embryos developed after injection of round spermatids into oocyte cytoplasm of the cynomolgus monkey (Macaca fascicularis). Hum Reprod 18, 1273–1280 (2003). 12773458 10.1093/humrep/deg212

[b57] Morgan P. M., Hutz R. J., Kraus E. M. & Bavister B. D. Ultrasonographic assessment of the endometrium in rhesus monkeys during the normal menstrual cycle. Biol Reprod 36, 463–469 (1987). 3555630 10.1095/biolreprod36.2.463

[b58] Mosdol G. Caesarean section in the Java monkey (Macaca irus)–a case report. J Small Anim Pract 17, 519–525 (1976). 823378 10.1111/j.1748-5827.1976.tb06995.x

