# Supplementary material for: Efficient production of cynomolgus monkeys with a toolbox of enhanced assisted reproductive technologies
Source: Sci Rep. 2016 May 13;6:25888. doi: 10.1038/srep25888 (PMC4865753; doi:10.1038/srep25888)
Supplement: Supplementary Information [file srep25888-s1.pdf]

Efficient production of cynomolgus monkeys with a toolbox of enhanced assisted reproductive technologies

Yunhan Ma[1,2,*] , Jiayu Li[1,*], Ge Wang[1,*] , Qiong Ke[3,*] , Sien Qiu[1], Liang Gao[1,4], Haifeng Wan[5], Yang Zhou[6] , Andy Peng Xiang[3] , Qunshan Huang[1] , Guoping Feng[6] , Qi Zhou[5] & Shihua Yang[1]

[1] College of Veterinary Medicine, Guangdong Provincial Key Laboratory of Prevention and Control for Severe Clinical Animal Diseases, South China Agricultural University, Guangzhou 510642, PR China;

[2] Shenzhen Key Lab of Neuropsychiatric Modulation and Collaborative Innovation Center for Brain Science, CAS Center for Excellence in Brain Science, Shenzhen Institutes of Advanced Technology, Chinese Academy of Sciences, Shenzhen 518055, PR China;

[3] Center for Stem Cell Biology and Tissue Engineering, Key Laboratory for Stem Cells and Tissue Engineering of Ministry of Education, Sun Yat-sen University, Guangzhou 510080, PR China;

[4] Blooming-spring biotechnology development Co., Ltd of Guangdong, Guangzhou 510940, PR China;

[5] State Key Laboratory of Reproductive Biology, Institute of Zoology, Chinese Academy of Sciences, Beijing 100101, PR China;

[6] McGovern Institute for Brain Research, Department of Brain and Cognitive Sciences, Massachusetts Institute of Technology, Cambridge, MA 02139, USA.

*These authors contributed equally to this work.

Correspondence and requests for materials should be addressed to G.P.F. (email: fengg@mit.edu) or Q.Z. (email: qzhou@ioz.ac.cn) or S.H.Y (email: yangsh@scau.edu.cn)

Parameter	Pregnant monkeys just before ET			Non-pregnant monkeys just before ET		
	Max	Min	Means ± s.e.m.	Max	Min	Means ± s.e.m.
Body weight (kg)	11.0	8.0	9.1 ±0.3	10.0	8.0	8.9 ±0.3
Age (year)	8.0	4.7	6.0 ±0.5	8.0	5.8	6.9 ±0.4
Volume ovary (cm^3)	9.9	3.7	6.7 ±1.1	9.6	2.7	5.5 ±1.1
Menstrual cycle（day）	15.0	11.0	13.1 ±0.7	15.0	11.0	14.4 ±0.6
Dominant follicle / Freshly ovulated follicle	1 / 9			1 / 9		
Embryos transferred	5.0	3.0	4.1 ±0.2	5.0	3.0	3.7 ±0.2
E2 (pg/mL)	646.0	31.5	157.9 ±122.1	653.5	32.8	177.8 ±119.1
P4 (ng/mL)	1.5	0.9	1.6 ±0.5	3.0	0.4	1.8 ±0.5
Uterus length (mm)	26.9	23.7	20.9 ±0.4	28.0	23.0	24.9 ±0.6
Endometrium length (mm)	21.8	11.5	19.2 ±1.0	21.2	12.5	18.9 ±1.1
Uterus height (mm)	25.7	13.8	21.1 ±1.3	23.3	13.4	18.9 ±1.1
Endometrium thickness (mm)	17.2	8.4	14.1 ±1.0	10.7	5.0	7.5 ±0.6**
Myometrium thickness (mm)	4.8	2.7	3.5 ±0.2	8.0	2.1	4.5 ±0.6
E/M	5.3	3.1	4.1 ±0.3	3.5	0.6	1.9 ±0.3**

Table S1. Parameters of Uterus and Ovaries at Embryo Transfer of Pregnant and Non-Pregnant Rhesus Recipients Received Embryos from the Same Fertilization Trial. Data are means ± s.e.m. with n=10 per group for the same experimental condition. P-values were analyzed with Student's t-test (nonparametric test, two tailed), ***P*< 0.01. Expect dominant follicle / freshly ovulated follicle with Fisher's exact test. Max, maximum value; Min, minimum value; ET, embryo transfer; E2, estradiol; P4, progesterone; E/M = endometrial to myometrial thicknesses.

Figure S1

A

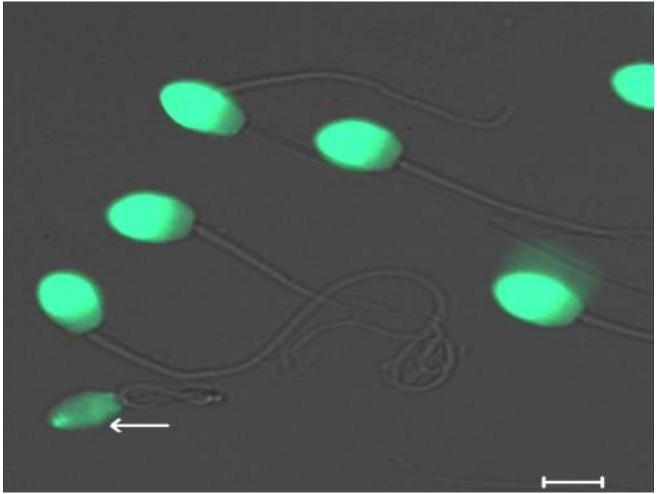

B

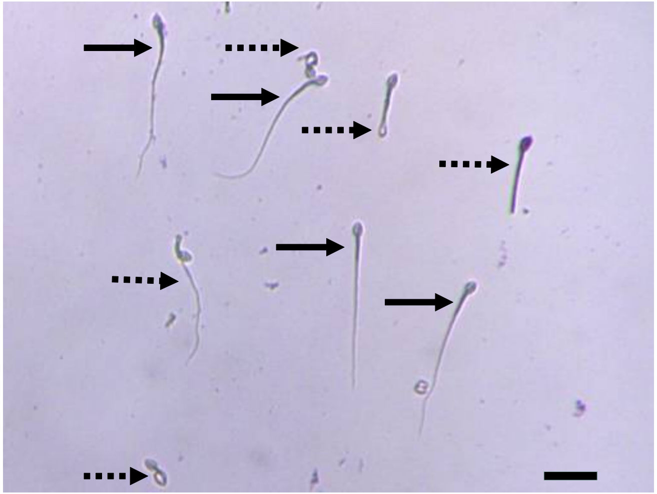

Figure S2

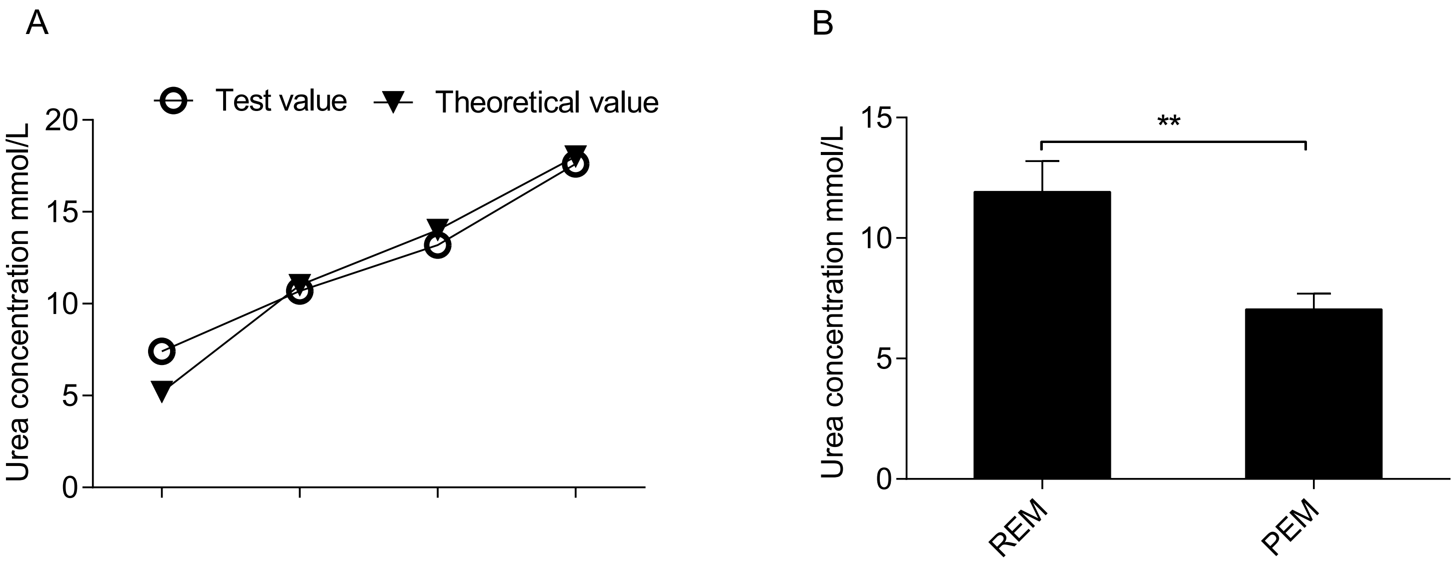
A
Test value
Theoretical value
Urea concentration mmol/L
20
15
10
5
0
B
Urea concentration mmol/L
15
10
5
0
**
REM
PEM

Figure S3

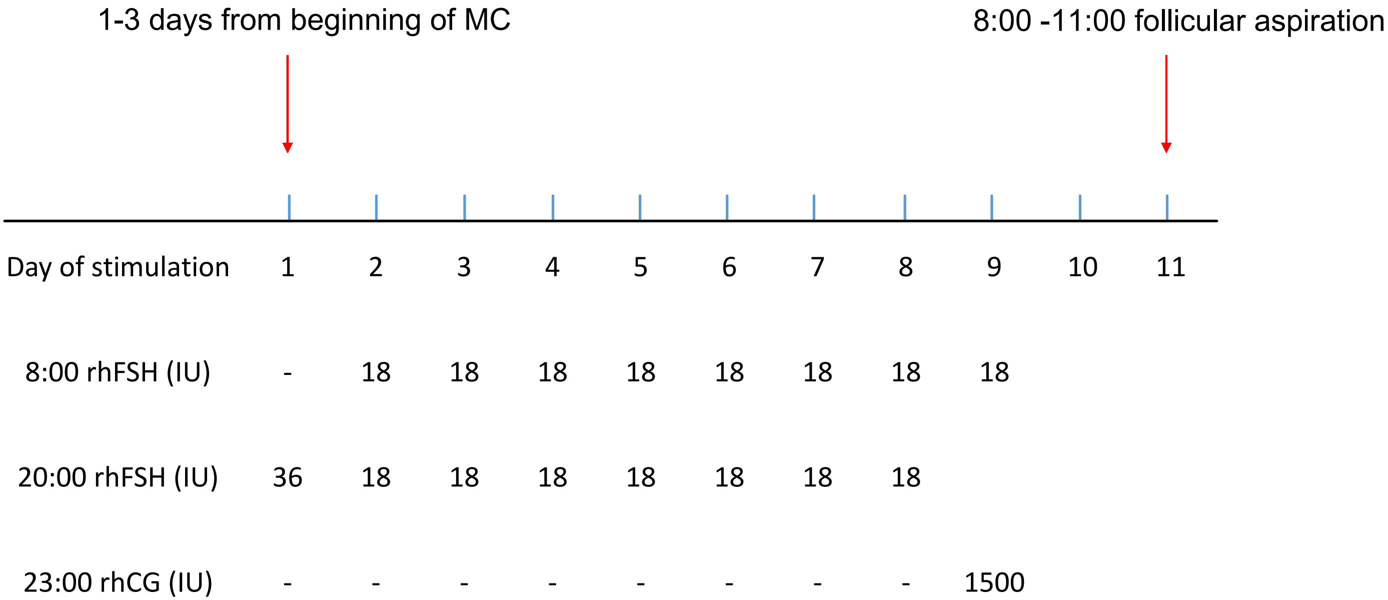
1-3 days from beginning of MC
8:00 -11:00 follicular aspiration
Day of stimulation
1
2
3
4
5
6
7
8
9
10
11
8:00 rhFSH (IU)
-
18
18
18
18
18
18
18
18
20:00 rhFSH (IU)
36
18
18
18
18
18
18
18
23:00 rhCG (IU)
-
-
-
-
-
-
-
-
1500

Figure S4

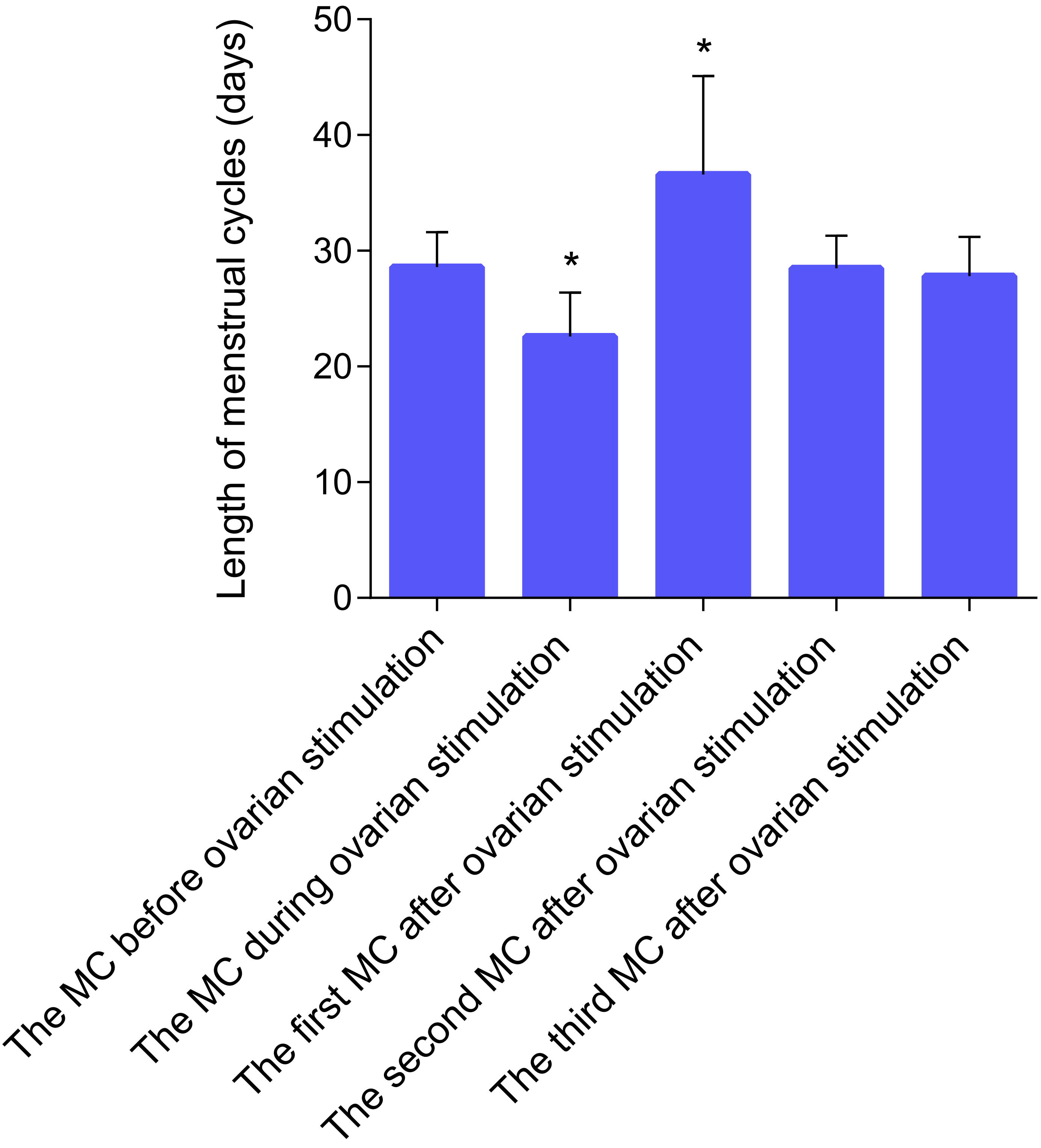
Length of menstrual cycles (days)
50
40
30
20
10
0
*
*
The MC before ovarian stimulation
The MC during ovarian stimulation
The first MC after ovarian stimulation
The second MC after ovarian stimulation
The third MC after ovarian stimulation

Figure S5

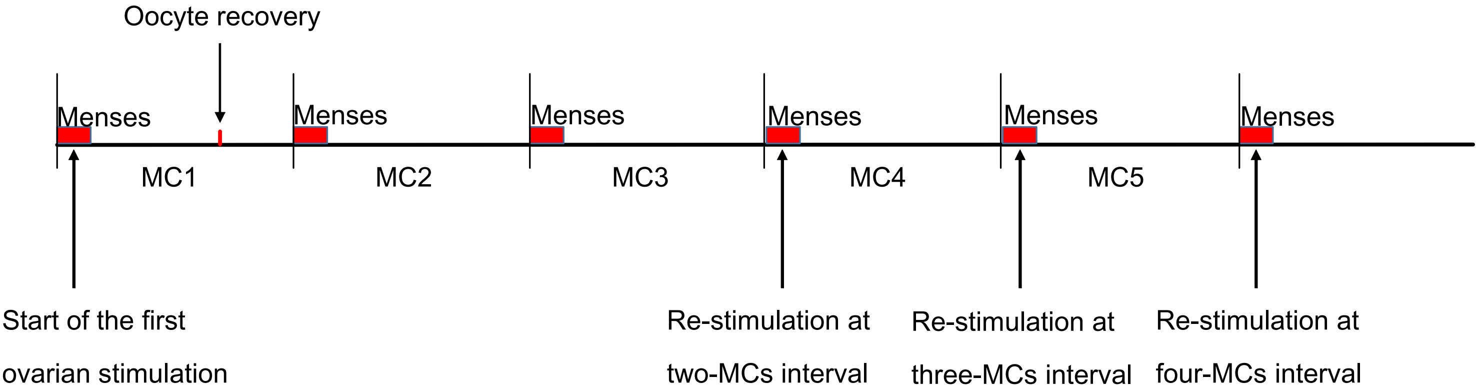
Oocyte recovery
Menses
Menses
Menses
Menses
Menses
Menses
MC1
MC2
MC3
MC4
MC5
Start of the first ovarian stimulation
Re-stimulation at two-MCs interval
Re-stimulation at three-MCs interval
Re-stimulation at four-MCs interval

Figure S6

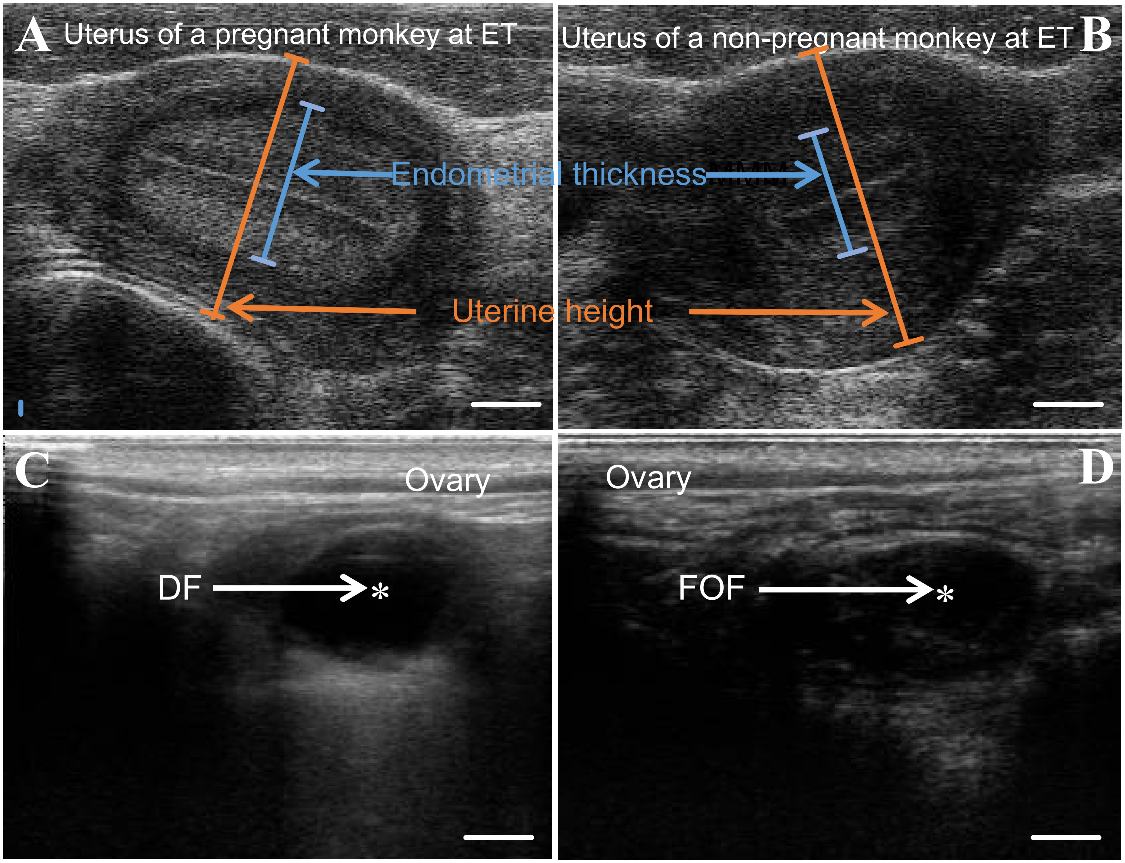

A
Uterus of a pregnant monkey at ET
Uterus of a non-pregnant monkey at ET
B
Endometrial thickness
Uterine height
C
Ovary
DF
*
Ovary
D
FOF
*

Figure S1. Cynomolgus Monkey Sperm. (A) Acrosome images, broken acrosomes marked with solid arrows, magnified at ×400 with an inverted microscope. (Scale bar, 5 μm). (B) Image of sperm morphology magnified at × 200 under an optical microscope. Normal sperm marked with solid arrow, sperm without tail or head, or short tail, and even sperm with sharp tail bend marked with dotted arrow. (Scale bar, 20 μm).

Figure S2. Measurement of Urea Concentration in Cynomolgus Monkey Semen. (A) Calibration curve of urea concentration in serum, the differences between test value and theoretical value is less than 15%. (B) Urea concentrations in fresh semen from REM and PEM. REM, rectal probe electrical stimulus method; PEM, penile probe electrical stimulation. Results are means ± s.e.m. Statistical analysis was performed with one-way analysis of variance. $^{**}P < 0.01$.

Figure S3. The Regimen of Ovarian Stimulation in Cynomolgus Monkeys.

Figure S4. Length of MCs Before and After Ovarian Stimulation. Data are means ± s.e.m. with n=8. Statistical analysis was done among different MCs with a generalized linear model repeated-measures $^{*}P < 0.05$.

Figure S5. Schematic of Repeated Ovarian Stimulation Separated at Intervals of Two, Three, or Four MCs after the Initial Stimulation with the Same Regimen. MCs, menstrual cycles.

Figure S6. Ultrasonic Images of Uterus and Ovary in Recipient Monkeys just before Embryo

Transfer. (A and B) Measurement of the endometrial thickness and uterine height (blue and red arrows, respectively) in a pregnant monkey and a non-pregnant monkey just before embryo transfer (Scale bar, 3 mm). (C and D) ovary state of recipient cynomolgus monkeys just before embryo transfer. A ovary with a dominant follicle (white arrow) and a ovary with a freshly ovulated follicle (white arrow) (Scale bar, 3 mm). ET, embryo transfer.
